# Boron-Doped Carbon Nanodots as a Theranostic Agent for Colon Cancer Stem Cells

**DOI:** 10.1021/acsomega.3c03154

**Published:** 2023-08-10

**Authors:** Sezgin Ozkasapoglu, Mehmet Gokhan Caglayan, Fatih Akkurt, Hilal Kabadayi Ensarioğlu, H. Seda Vatansever, Huseyin Celikkan

**Affiliations:** † Turkish Nuclear Energy and Mineral Research Agency (TENMAK), Boron Research Institute (BOREN), Ankara 06520, Turkey; ‡ Faculty of Pharmacy, Department of Analytical Chemistry, Ankara University, Ankara 06560,Turkey; § Faculty of Engineering, Department of Chemical Engineering, Gazi University, Ankara 06570, Turkey; ∥ Faculty of Medicine, Department of Histology and Embryology, Manisa Celal Bayar University, Manisa 45030, Turkey; ⊥ DESAM Institute, Near East University, Mersin 10, Turkey; # Science Faculty, Department of Chemistry, Gazi University, Ankara 06500, Turkey

## Abstract

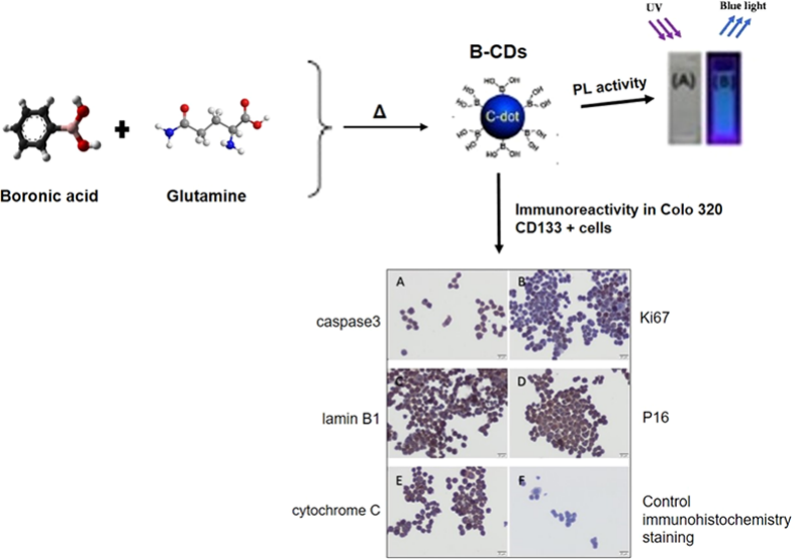

Carbon nanodots have drawn a great deal of attention due to their green and expedient opportunities in biological and chemical sciences. Their high fluorescence capabilities and low toxicity for living cells and tissues make them excellent imaging agents. In addition, they have a fluorimetric response against inorganic and organic species. Boron-doped carbon nanodots (B-CDs) with high fluorescence yield were produced from phenylboronic acid and glutamine as boron and carbon sources, respectively, by a hydrothermal method. First, the effects of the temperature on their fluorescence yield and the structural characteristics of B-CDs were investigated. Second, their cytotoxicity and cell death and proliferation behaviors were examined. The cytotoxicity was evaluated by the MTT assay. The cellular properties were evaluated with the distribution of caspase 3, Ki67, lamin B1, P16, and cytochrome *c* after the indirect immunoperoxidase technique. After the MTT assay, 1:1 dilution of all applicants for 24 h was used in the study. After immunohistochemical analyses, the application of B-CDs synthesized at 230 °C did not change control cell (Vero) proliferation, and also apoptosis was not triggered. Colo 320 CD133+ and CD133– cell-triggered apoptosis and cellular senescence were found to be synthesis temperature dependent. In addition, Colo 320 CD133– cells were affected relatively more than CD133+ cells from B-CDs. While B-CDs did not affect the control cells, the colon cancer stem cells (Colo 320 CD133+) were affected in a time-dependent manner. Therefore, the use of the synthesized B-CD product may be an alternative method for controlling or eliminating cancer stem cells in the tumor tissue.

## 1 Introduction

Carbon nanodots (CDs) were first discovered during the purification of single-walled carbon nanotubes in 2004.^1^ Since their discovery, they have attracted much research interest. Because of their excellent optical properties, biocompatibility, water solubility, photostability, and ease of cost-effective synthesis, they have attracted great attention in a wide range of chemical and biomedical applications from sensing, bioimaging, phototherapy, and catalysis to energy-related applications.

CDs were highly preferred for diagnostic purposes in living organisms because of their low toxicity, good water solubility, and high biocompatibility. Cancer is one of the most dangerous diseases with over 10 million new cases each year.^2^ In the literature, a number of publications about the potential application of CDs in early cancer diagnostics were reported.^3,4^ It is known that folic acid (FA) has a potential to bind and penetrate into cancer cells due to plenty of folate receptors (FR) on various cancer cell membranes. For example, the surface of carbon nanodots was coated with FA to target MCF-7 breast cancer cells.^5^ Central nervous system (CNS) tumors are another dangerous diseases that can cause death. Especially, glioma, which is a brain tumor, is the most common and fatal brain tumor in humans.^6^ Zheng et al. synthesized CDs with a self-targeting ability using d-glucose and l-aspartic acid as precursors. These CDs showed high selectivity and enhancement of imaging quality in brain glioma cells.^7^ The blood–brain barrier (BBB) is a barrier system that is vital for the central nervous system.^8^ Drug delivery to the brain is still a major problem because of the BBB, which has strict junctions between endothelial cells, blocking the crossing of the therapeutic agents to pathological tissues in the brain. However, the strict junctions in the BBB have a gap in the range of 4–6 nm. Thus, the nanoparticles with a size below 4 nm could cross the BBB through such gaps.^9^ Nitrogen-doped CDs synthesized via the hydrothermal route by Lu’s group have a small size of approximately 2.6 nm.^10^ These CDs have an enhanced ability to cross the BBB due to their small size.

CDs have been synthesized through various approaches, which can be divided into two categories: top–down and bottom–up synthesis methods.^11^ Compared to top–down methods, bottom–up methods are more advantageous in terms of precursors and carbonization methods that can be selected.^12^ “Bottom–up” approaches generally utilize different methods, like carbonization of carbohydrates, self-assembly of polycyclic aromatic hydrocarbons, and organic synthesis from small molecules under a range of different reaction conditions including hydrothermal/solvothermal, microwave-assisted, and ultrasound-supported synthetic conditions.^1^ Among these, the hydrothermal route stands out as a reliable and repeatable method.^11^

Doping the CDs with other elements such as boron, nitrogen, sulfur, phosphorus, etc. is an influential method for generating the optical properties in CDs. Because boron’s ionic radius is similar to that of carbon, it is possible to effectively modulate the properties of CQDs after doping with boron.^13^ Moreover, recent studies have indicated that doping could extensively increase the quantum yield of CDs.^14^

In this study, fluorescent boron-doped CDs (B-CDs) were obtained through a hydrothermal method using phenylboronic acid and l-glutamine as the precursors. The effect of temperature on their fluorescence yield and the structural characteristics of B-CDs were investigated. The as-synthesized CDs emitted blue fluorescence in a quantum yield of 2.9%. Cytotoxicity, cell death, and proliferation behavior of B-CDs were examined. Cytotoxicity was evaluated by the MTT assay. The cellular properties were evaluated from the distribution of caspase 3, Ki67, lamin B1, P16, and cytochrome *c* after an indirect immunoperoxidase technique.

## 2 Results and Discussion

**Figure 1:**
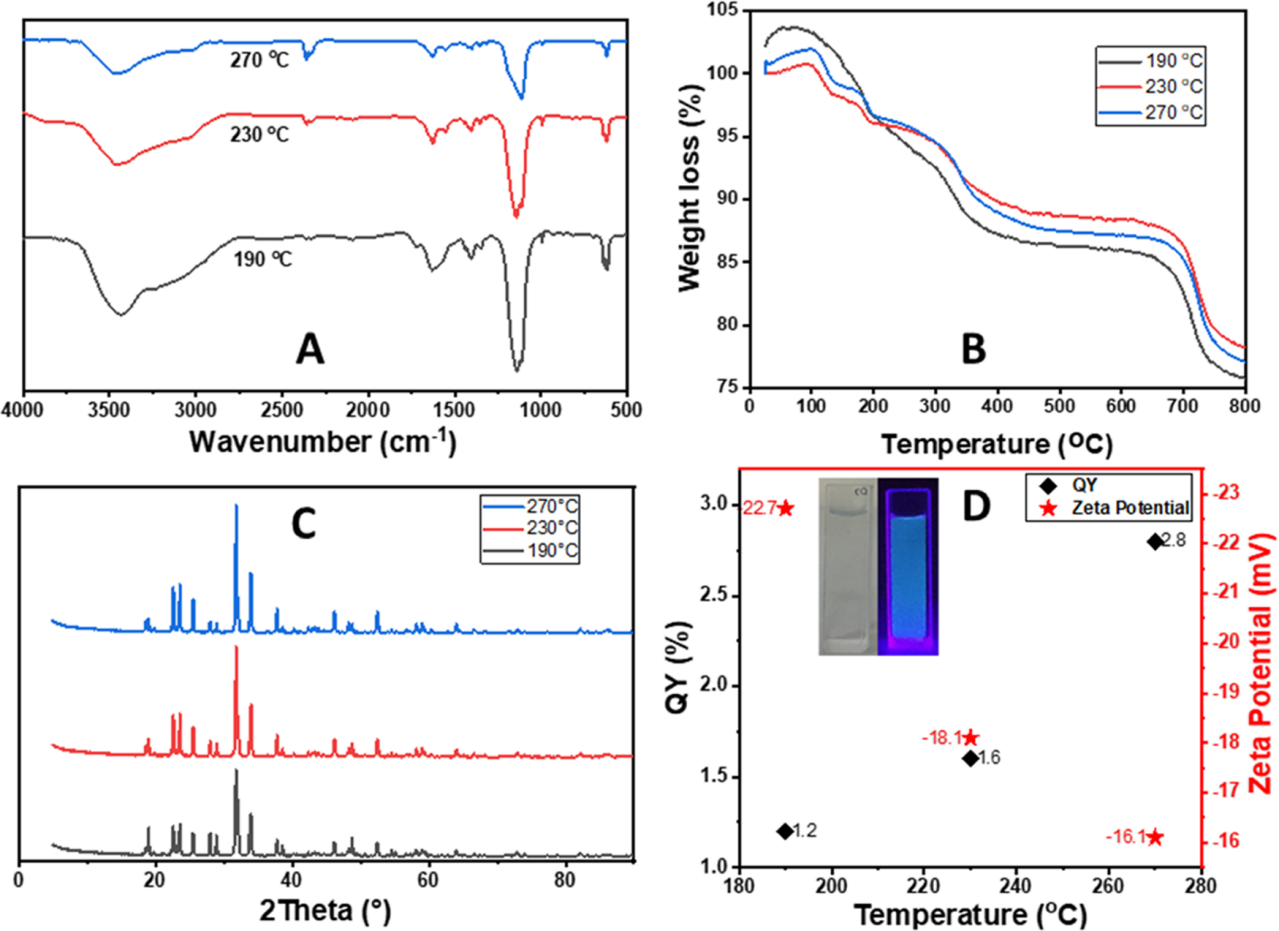
FTIR spectra (A), thermograms (B), X-ray diffraction patterns (C), ζ potentials, and QYs (D) of B-CDs synthesized at 190, 230, and 270 °C. Inset of D: The photographs of B-CDs synthesized at 270 °C under daylight (left panel) and under UV light (395 nm) (right panel).

The structural characterization of B-CDs was performed before their optical characterization. The surface functional groups and bond structure of B-CDs synthesized at three different temperatures, 190, 230, and 270 °C, were examined by Fourier transform infrared (FTIR) analysis. When the spectrum in Figure 1A was examined, it appeared that the peaks were not temperature dependent. The spectrum in Figure 1A shows a broad band at around 3500 cm^–1^ assigned to B–NH or B–OH stretching. The peak at around 1630 cm^–1^ was assigned to the C=O vibration. The peak at 1400 cm^–1^ showed a band of B–N stretching. The stretching bands of B–C, C–C, and C–N bonds should be responsible for the strong peak at around 1130 cm^–1^.^15^ The B–O stretching band was confirmed by the appearance of the peak at 995 cm^–1^,^16,17^ and the peak at around 620 cm^–1^ confirmed the presence of BO_2_ from out of plane bending. Finally, FTIR analysis revealed that the boron atom was inserted into the B-CDs, and the B-CDs were highly hydrophilic and well dispersed in water due to the polar functional groups on their surfaces. Thermal behaviors of B-CDs were investigated by a thermogravimetric analysis (TGA) analysis. The thermograms showed that the loss of mass was nearly 25% in all three products up to 800 °C in Figure 1B. This confirmed that B-CDs were thermally stable and acquired an inorganic character in high-temperature synthesis. The loss of mass was associated with the divergence of functional groups from the structure as the temperature increased.^18^ The crystal structure of B-CDs was investigated by the X-ray diffraction (XRD) technique. It was seen that B-CDs had high crystallinity independent of temperature due to synthesis conditions such as solvent type, temperature, and pH.^19^ The formation of high crystallinity in B-CDs was associated with the presence of sulfate in the reaction medium.

ζ potential was a significant parameter to evaluate the stability of B-CDs and indicated the penetration of B-CDs from the cell membrane.^20^ ζ potential measurements of B-CDs were performed at neutral pH. As seen in Figure 1D, the surfaces of B-CDs were negatively charged due to the presence of carboxyl groups on their surfaces. This behavior endowed B-CDs with the ability to disperse well in water and promoted their usability in biological applications.^20^ As the temperature was increased, it was seen that the ζ potential became less negative because of the removal of carboxyl and hydroxyl groups, which provided negative charges to B-CDs. As it is known, the ζ potential higher than |30 mV| demonstrates good stability in the water toward aggregation due to the electrostatic repulsion of the particles.^21^

**Figure 2:**
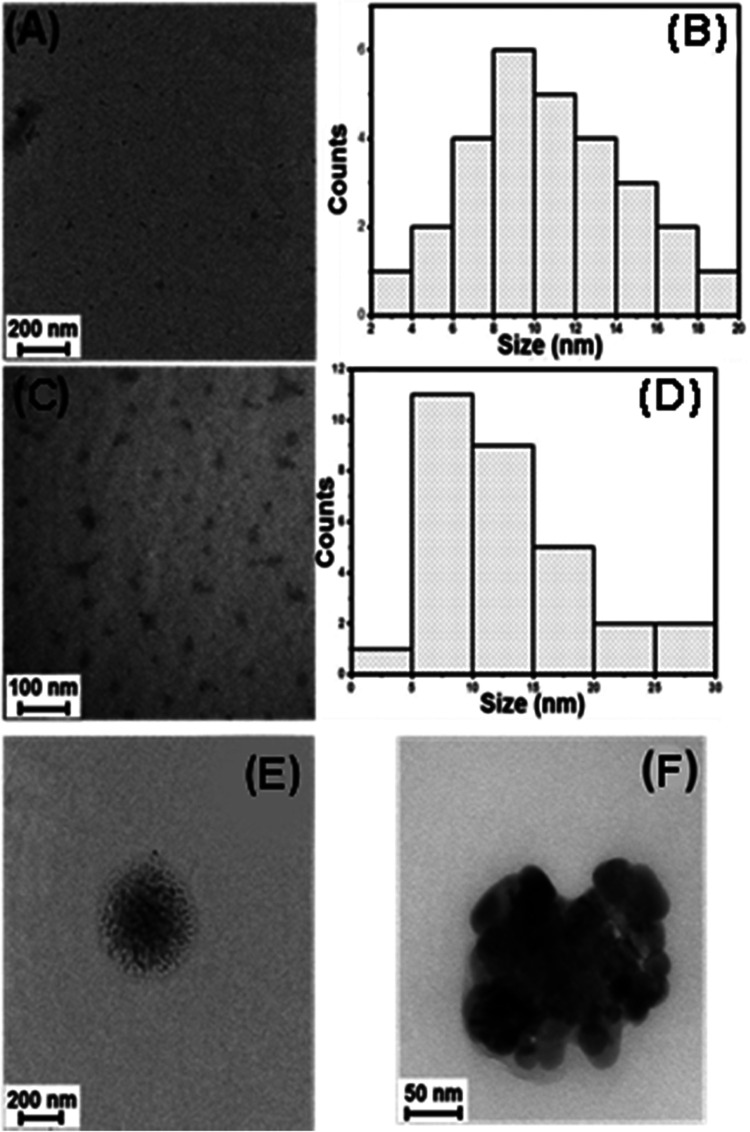
Transmission electron micrographs of B-CDs synthesized at (A) 190 °C, (C) 230 °C, (E) and (F) 270 °C. The histograms for their size distributions of (B) 190 °C and (D) 230 °C.

Transmission electron micrographs of B-CDs are depicted in Figure 2. Although the histograms from the micrographs obtained for B-CDs were synthesized at 190 and 230 °C, they could not be obtained for the product at 270 °C due to aggregation in Figure 2. It was seen from the micrographs that, principally, the particle size and aggregation of B-CDs increased with temperature. The particle ratio of B-CDs smaller than 10 nm was 42 and 40% at synthesis temperatures of 190 and 230 °C, respectively. The remaining B-CDs were largely under 20 nm, as seen in Figure 2B,C. The high aggregation in B-CDs synthesized at 270 °C was attributed to the loss of functional groups on the surface that provided dispersion stability in water, which is consistent with the ζ potential results.^22^

Carbon, hydrogen, nitrogen, and sulfur contents of B-CDs at 190, 230, and 270 °C are listed in Table 1. Carbon and nitrogen contents of B-CDs were decreased by increasing reaction temperature as expected due to the release of carbonaceous and nitrogenous gases. Interestingly, the sulfur content of B-CDs was observed regardless of the synthesis temperature because of the sulfating effect of the products.

**Table 1:** Elemental Analysis of B-CDs Synthesized at 190, 230, and 270 °C^a^

temperature (°C)	C (%)	H (%)	N (%)	S (%)	B (%)
190	10.1	1.09	2.25	18.4	0.28
230	8.27	1.12	1.83	19.2	0.34
270	9.26	1.21	1.73	18.5	0.25

a Boron contents were determined by ICP-MS.

X-ray photoelectron spectroscopy (XPS) was performed to determine the chemical binding of C, N, O, S, and B atoms in B-CDs. The core level XPS spectra of C 1s, N 1s, O 1s, S 2p, and B 1s for B-CDs synthesized at 190 °C, 230, and 270 °C are presented in Figures S2, S3, and S4, respectively. Locations of chemically shifted C 1s, B 1s, N 1s, O 1s, and S 2p are listed in Tables 2, 3, S1, S2, and S3 with their percentages, respectively. In Table 2, obtained C 1s core level of B-CDs synthesized at various temperatures, C–H, C–C, C=C, C–N, C–OH, C-ON, C=O, C–SO_3_^–^, and O–C=O are presented as expected chemical shifts of the C 1s core level by XPS in agreement with the literature.^23,24^ In Table 2, it can be seen that increasing temperature was highly effective for sulfonation and C–N bond breakage of B-CDs, in which 230 °C was interestingly found as the optimum temperature because of the gasification process of carbon. In addition, the oxidation behavior of B-CDs was observed by the increase of oxygen functionalities of carbon atoms, as in Table 2. The N 1s core level of B-CDs is listed in Table S1, and N–B formation was proven by XPS measurements. In addition, the group of alkyl ammonium was observed as well as C–N groups in the organic matrix in Table S1. The chemical environments of the O 1s core level from XPS measurements are listed in Table S2, and a moderate temperature of 230 °C was found to be critical to obtain the oxygen-rich functionality of B-CDs. The increase in aromatic C–O–C and the decrease in aliphatic C–O–C with increasing synthesis temperature of B-CDs were compatible with the strength of these structures for their decomposition. The sulfate ratio of B-CDs decreased with increasing synthesis temperature, and it was in agreement with the decrease of ζ potentials in Figure 1D due to decreased charges on the B-CD surface. Boron contents in CDs are given in Table 3 obtained from B 1s core level measurements by XPS. In Table 3, B–C, B–H, B–O, and B–N were observed in B-CD structures, and B–N bond formation was accepted as evidence for the chemical inclusion of boron into CDs to obtain B-CDs. Interestingly, not only sulfo groups but also sulfate groups were observed.

**Table 2:** Binding Energies and Percentages of C 1s for B-CDs Synthesized at 190, 230, and 270 °C

	synthesis temperature of B-CDs					
	190 °C		230 °C		270 °C	
C 1s	B.E. (eV)	%	B.E. (eV)	%	B.E. (eV)	%
C=C	284.0	2.38	283.9	1.46	284.0	1.60
C–C, C–H	285.0	14.5	285.0	14.2	285.0	21.2
C–N	285.7	24.2	285.7	17.4	285.8	17.2
C–O–H, C–O–N	286.3	22.0	286.5	30.4	286.5	25.5
C=O	287.1	13.6	287.4	13.1	287.5	7.79
C–SO_3_^–^	288.0	7.27	288.5	14.2	288.5	9.94
O–C=O	289.6	16.1	289.7	15.7	289.4	16.9

**Table 3:** Binding Energies and Percentages of B 1s for B-CDs Synthesized at 190, 230, and 270 °C

	synthesis temperature of B-CDs					
	190 °C		230 °C		270 °C	
B 1s	B.E. (eV)	%	B.E. (eV)	%	B.E. (eV)	%
B–C, B–H	188.2	24.6	188.2	14.0	187.5	25.7
B–N	190.7	19.8	190.5	27.0	190.2	22.0
B–O	192.9	55.6	193.5	59.0	193.0	52.3

**Figure 3:**
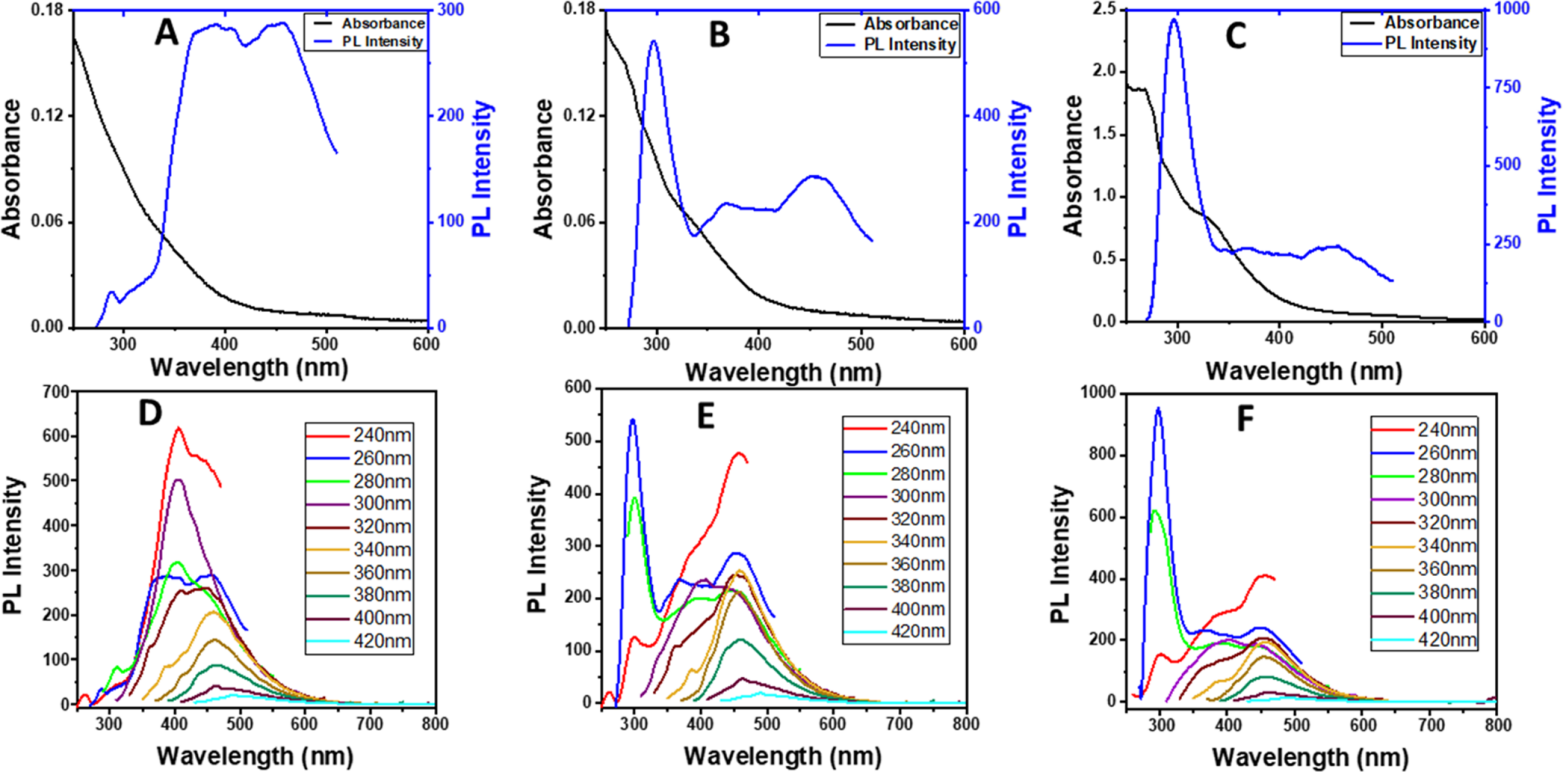
UV–vis absorption (black line) and PL emission (blue line) of B-CDs synthesized at 190 °C (A), 230 °C (B), and 270 °C (C), and PL spectra of B-CDs synthesized at 190 °C (D), 230 °C (E), and 270 °C (F) under various excitation wavelengths (starting from 240 nm in 20 nm increments below).

The UV–vis and photoluminescence (PL) spectra of the as-synthesized B-CDs at 190, 230, and 270 °C are presented in Figure 3. The spectra of B-CDs synthesized at 230 and 270 °C showed two weak and more apparent peaks at around 265 and 330 nm, respectively. The peak at around 265 nm was attributed to the characteristic aromatic π–π* (C=C) transition of B-CDs because of sp^2^ hybridization.^14^ The peak at around 330 nm was ascribed to n-π* of C=O functional groups on the B-CD structure.^25^

Although the PL mechanism of CDs is still unclear, it is surveyed in the literature with two theories explaining the origin of the PL mechanism, which are intrinsic state effect and defective state emission.^11^ The spectra in Figure 3 show that as the excitation wavelength increased from 240 to 320 nm in 20 nm increments, PL emissions shifted to longer wavelengths, especially for B-CDs synthesized at 230 and 270 °C, so this behavior was one of the characteristics of CDs.

### 2.1 Cytotoxicity of B-CDs

**Figure 4:**
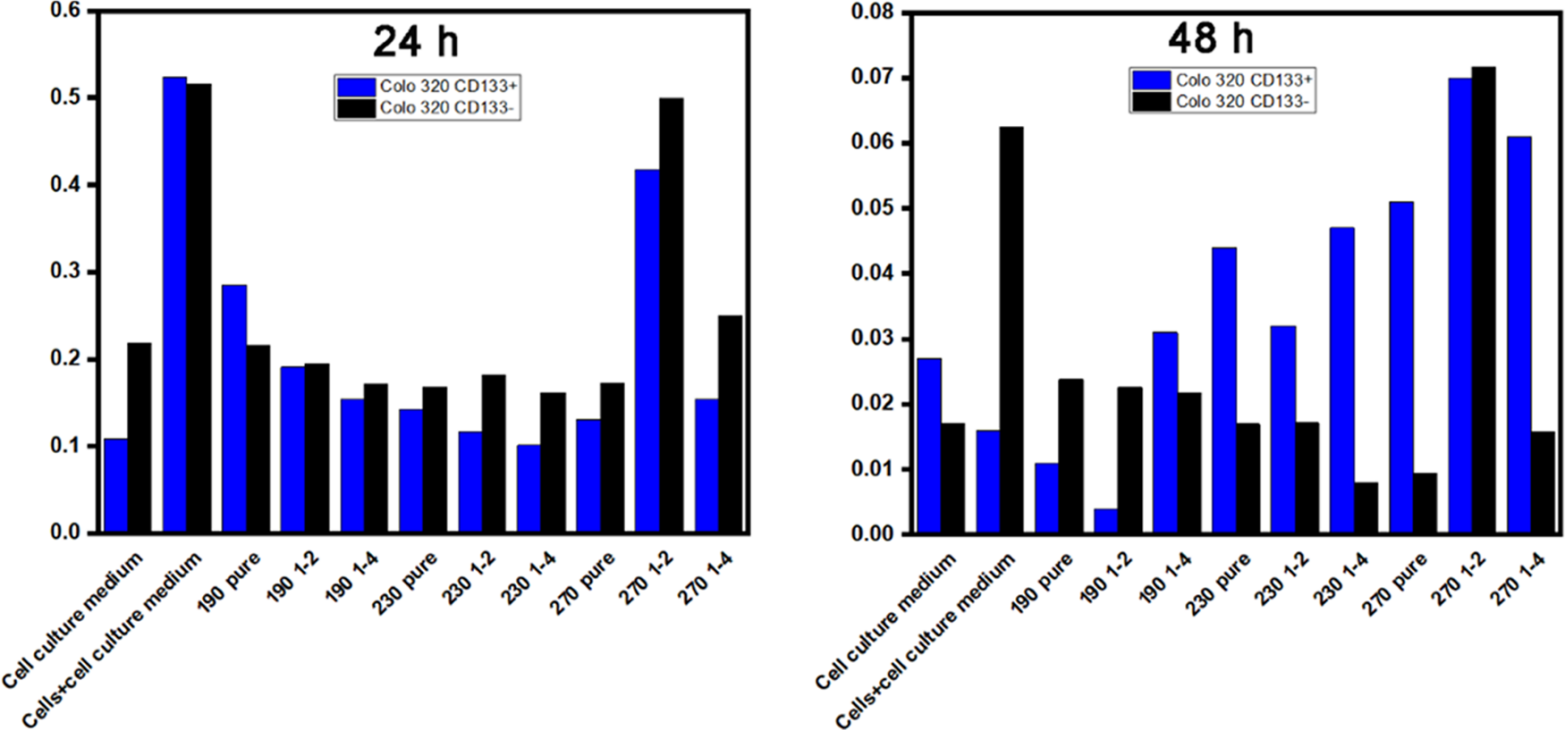
Cytotoxicity values of B-CDs synthesized at 190, 230, and 270 °C in Colo 320 CD133+ and CD133– cells after 24 and 48 h.

Since the 48-h application of B-CDs was toxic to the cells as demonstrated by the result of MTT analyses for both Colo 320 CD133+ and CD133– cells, 1:1 dilution of all applicants for 24 h was used in the study (Figure 4).

### 2.2 Immunohistochemistry (Vero Cells)

Immunoreactivities of caspase 3, Ki67, lamin B1, P16, and cytochrome *c* in Vero cells after 24 h of glutamine administration at B-CDs synthesized at 190, 230, and 270 °C are presented in Figures S5, S6, and S7, respectively. After the application of glutamine 190 °C for 24 h in Vero cells, which was determined as the control, while caspase 3 (Figure S5A), Ki67 (Figure S5B), P16 (Figure S5D), and cytochrome *c* (Figure S5E) immunoreactivities were weak (+) in all cells, strong (+++) immunoreactivity of lamin B1 (Figure S5C) was detected. After 24 h of glutamine 230 °C application in Vero cells, caspase 3 (Figure S6A) and P16 (Figure S6D) immunoreactivities were negative. Weak (+) immunoreactivities of Ki67 (Figure S6B) and cytochrome *c* (Figure S6E) were observed. In contrast to that, strong (+++) immunoreactivity of lamin B1 was detected in all cells (Figure S6C). After 24 h of glutamine 270 °C application in Vero cells, caspase 3 (Figure S7A),

Ki67 (Figure S7B), and cytochrome *c* (Figure S7E) immunoreactivities were weak (+) in all cells. While P16 immunoreactivity was negative in all cells (Figure S7D), and strong (+++) immunoreactivity of lamin B1 (Figure S7C) was observed. The absence of staining in the control immunohistochemistry (Figures S5–S7F) indicates that the immunoreactivities are specific.

### 2.3 Immunohistochemistry (Colo 320)

**Figure 5:**
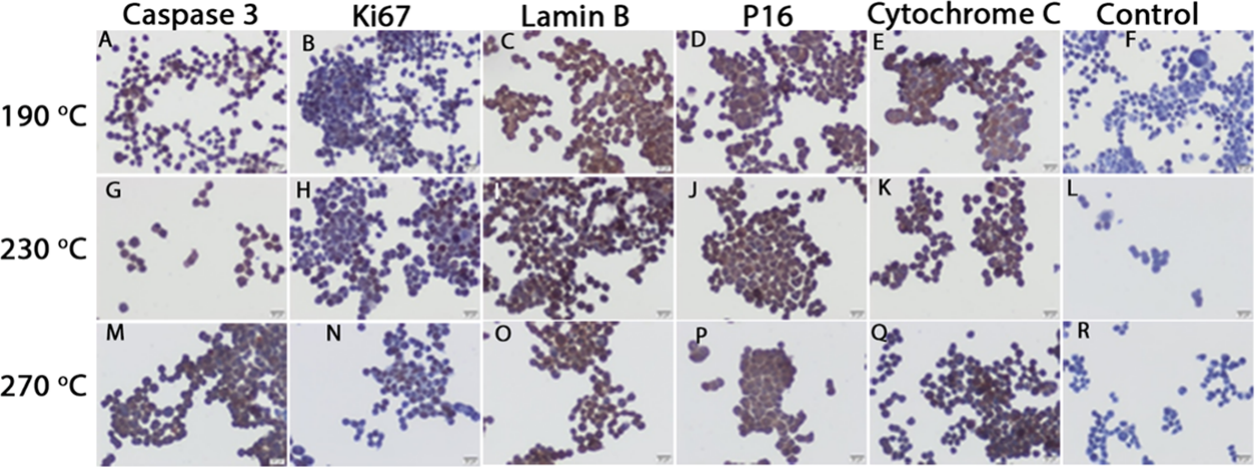
Caspase 3 (A, G, M), Ki67 (B, H, N), lamin B1 (C, I, O), P16 (D, J, P), and cytochrome *c* (E, K, O) immunoreactivity in Colo 320 CD133 + cells after 24 h of treatment with B-CDs synthesized at 190 °C, 230, and 270 °C respectively. Control immunohistochemical staining (F, L, R). Scale bars: 20 μm.

As a result of the application of B-CD synthesized at 190 °C for 24 h in Colo 320 CD133+ cells, caspase 3 immunoreactivity was found to be weak (Figure 5A), while Ki67 immunoreactivity was negative (Figure 5B). Immunoreactivities of lamin B1 (Figure 5C), P16 (Figure 5D), and cytochrome *c* (Figure 5E) were detected as moderate.

Application of B-CDs synthesized at 190 °C triggered apoptosis and cellular senescence with the observation of cytochrome *c* and P16 immunoreactivities in Colo 320 CD133+ cells, and the observation of lamin B1 immunoreactivity suggested that there was a decrease in cellular senescence. The fact that caspase 3 immunoreactivity was lower than the others suggested that apoptosis started with an increase in cytochrome *c*, but it could not cause an increase in caspase 3 in all cells, suggesting that the reason for this may be the 24-h application. The absence of staining in Ki67 indicates that glutamine 190 °C did not induce proliferation in Colo 320 CD133+ cells.

The effect of the application of B-CDs synthesized at 230 °C for 24 h in Colo 320 CD133+ cells was similar to those of B-CDs synthesized at 1900C, but weak and moderate caspase 3 immunoreactivity was detected (Figure 5G). While Ki67 immunoreactivity was negative (Figure 5H), lamin B1 (Figure 5I), P16 (Figure 5J), and cytochrome *c* (Figure 5K) immunoreactivities were moderate. In addition, some cells have strong intensity of P16 (Figure 5J). It was thought that the application of B-CDs synthesized at 230 °C triggered apoptosis in the presence of cytochrome *c* and caspase 3 immunoreactivities in Colo 320 CD133+ cells but did not trigger proliferation with negative Ki67 immunoreactivity. The fact that lamin B1 shows less cellular senescence than P16 suggested that B-CD synthesized at 230 °C also triggered senescence in Colo 320 CD133+ cells.

Application of B-CDs synthesized at 190 °C triggered apoptosis and cellular senescence with the observation of cytochrome *c* and P16 immunoreactivities in Colo 320 CD133+ cells, and the observation of lamin B1 immunoreactivity suggested that there was an increase in cellular senescence. The fact that caspase 3 immunoreactivity was lower than the others suggested that apoptosis started with an increase in cytochrome *c*, but it could not cause an increase in caspase 3 in all cells, suggesting that the reason for this may be the 24-h application. The absence of staining in Ki67 indicates that 190 °C glutamine did not induce proliferation in Colo 320 CD133+ cells.

The effect of the application of B-CDs synthesized at 230 °C for 24 h in Colo 320 CD133+ cells was similar to those of B-CDs synthesized at 190 °C, but weak and moderate caspase 3 immunoreactivity was detected (Figure 5G). While Ki67 immunoreactivity was negative (Figure 5H), lamin B1 (Figure 5I) and P16 (Figure 5J), and cytochrome *c* (Figure 5K) immunoreactivities were moderate. In addition, some cells have strong intensity of P16 (Figure 5J).

It was thought that the application of B-CDs synthesized at 230 °C triggered apoptosis with the presence of cytochrome *c* and caspase 3 immunoreactivities in Colo 320 CD133+ cells but did not trigger proliferation with negative Ki67 immunoreactivity. The fact that lamin B1 was less than P16 in cellular senescence suggested that the application of B-CD synthesized at 230 °C also triggered senescence in Colo 320 CD133+ cells.

Although the application of B-CD synthesized at 270 °C had higher caspase 3 and P16 immunoreactivities in Colo 320 CD133+ cells compared to glutamine 190 and 230 °C applications, it was concluded that apoptosis and cellular senescence were triggered in the cells. In addition, Ki67, which is a proliferation marker, showed weak immunoreactivity (Figure 5). Control immunohistochemistry staining is also shown in Figure 5F,L,R.

**Figure 6:**
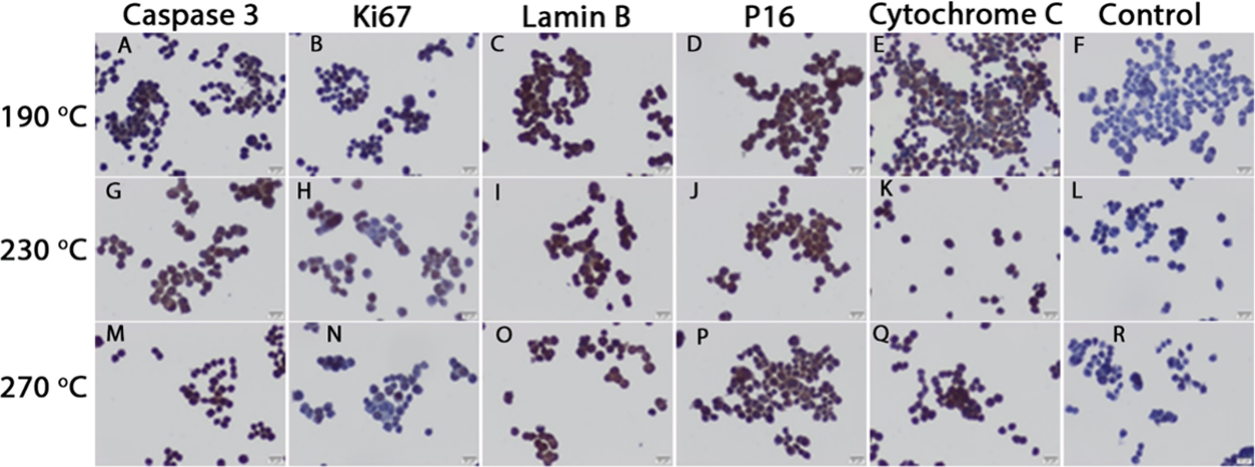
Caspase 3 (A, G, M), Ki67 (B, H, N), lamin B1 (C, I, O), P16 (D, J, P), and cytochrome *c* (E, K, O) immunoreactivity in Colo 320 CD133– cells after 24 h of B-CD treatment synthesized at 190, 230, and 270 °C, respectively. Control immunohistochemical staining (F, L, R). Scale bars: 20 μm.

After 24-h application of B-CDs synthesized at 190 °C treatment in Colo 320 CD133– cells, caspase 3 immunoreactivity was very weak (Figure 6A) and Ki67 immunoreactivity was negative (Figure 6B). Lamin B1 (Figure 6C) and P16 (Figure 6D) immunoreactivities were similar in Colo 320 CD133– cells and moderate. cytochrome *c* immunoreactivity was moderate and rarely strong (Figure 6E).

This indicates that the application of B-CD synthesized at 190 °C causes an increase in cytochrome *c* in Colo 320 CD133– cells but triggers apoptosis due to low caspase 3 immunoreactivity but does not completely terminate. However, it can be suggested that cellular senescence starts with an increase in P16 immunoreactivity, but with the presence of lamin B1 immunoreactivity, a balance is tried to be established between cellular senescence and survival.

After 24-h application of B-CD synthesized at 230 °C in Colo 320 CD133– cells, caspase 3 (Figure 6G), lamin B1 (Figure 6I), and P16 (Figure 6J) immunoreactivities were found to be moderate. Ki67 immunoreactivity was weak (Figure 6H). cytochrome *c* immunoreactivity was strong (Figure 6K).

It was thought that the application of B-CDs synthesized at 230 °C compared to that at 190 °C triggered apoptosis in Colo 320 CD133– cells due to the increase in both cytochrome *c* and caspase 3. It gave the impression that cellular senescence in Colo 320 CD133– cells started with an increase in P16 immunoreactivity similar to B-CD synthesized at 190 °C application, but it was tried to maintain balance with the presence of lamin B1 immunoreactivity.

In Colo 320 CD133– cells, after application of B-CDs synthesized at 270 °C, strong caspase 3, lamin B1, and P16; moderate cytochrome *c* and weak Ki67 immunoreactivities were detected (Figure 6). Control immunohistochemistry staining is also shown in Figure 6F,L,R.

It was observed that apoptosis in Colo 320 CD133– cells was higher with the application of B-CDs synthesized at 270 °C compared to others, and cellular senescence was similar.

## 3 Conclusions

Carbon nanodots are attracting attention due to a variety of features they have, such as biocompatibility, low toxicity, and controllable fluorescence response through their elemental compositions and synthesis conditions. Their high fluorescence capabilities enable carbon nanodots to become excellent imaging and theranostic agents for living cells and tissues. In this work, apoptosis and senescence were triggered in colon cancer stem cells. In addition, the application of B-CDs synthesized at 230 °C was the best for triggering cell death and cellular senescence in Colo 320 CD133+ cells. Therefore, this administration of B-CDs (of 230 °C) may be utilized to control the influence of colon cancer stem cells balance between the cell survival of either apoptosis or senescence.

## 4 Materials and Methods

### 4.1 Chemicals

Phenylboronic acid and glutamine as precursors were obtained from Sigma Aldrich. The other chemicals such as H_2_SO_4_, Na_2_CO_3_, Na_2_SO_4_, and NaOH were obtained from various suppliers, and they were of ACS grade. The dialysis tube was obtained from Spectra/Por at 2 kDa of MW. Nitrocellulose filter paper with 0.22 μm pore size was obtained from Sartorius. The chemicals used in the assay and immunohistochemistry experiments are also mentioned in the relevant sections. In all experiments, deionized water was used at 18.2 MΩ.

### 4.2 Instruments and Apparatus

UV–vis absorption spectra were obtained using an Agilent/Cary 60 spectrometer. Fluorescence measurements were performed on an Agilent/Cary Eclipse spectrometer. X-ray diffraction (XRD) measurements were carried out on a PANalytical/Empryean diffractometer with Cu Kα radiation (λ = 1.5406 Å). Fourier transform infrared spectroscopy spectra were obtained on a Varian/660 IR spectrometer. X-ray photoelectron spectroscopy (XPS) measurements were performed using a Thermo Scientific/K-Alpha XPS spectrometer using Al Kα radiation (λ = 8.3386 Å). Transmission electron micrographs (TEM) were obtained using an FEI/Tecnai G^2^ Spirit Biotwin electron microscope using an acceleration voltage of 120 kV. The amount of C, N, S, and H elements in CDs was determined using a Thermo Scientific/S2000 elemental analyzer, while boron content of CDs was determined using a PerkinElmer ELAN 9000 ICP-MS after homogenization of the samples in nitric acid. Thermal characterization was carried out using a Netzsch/STA 449 F3 thermal analyzer device. ζ potentials of the B-CDs were obtained using a Malvern/Zetasizer Nano ZSP.

### 4.3 Synthesis of B-Doped Carbon Nanodots (B-CDs)

B-CDs were synthesized by the hydrothermal route using phenylboronic acid (PBA) and l-glutamine (GLU) as the starting materials. Briefly, 0.25 g of PBA and GLU were placed in a Teflon-lined stainless steel autoclave. Approximately, 25 mL of 1.0 M H_2_SO_4_ was added to the starting materials in the autoclave. Then, the reactor was transferred to an oven and heated at three different temperatures (190, 230, and 270 °C) for 2 h. The reactor was allowed to cool down to room temperature. After cooling to room temperature, the as-prepared B-CD solution was filtered using a 0.22 μm filter paper. pH was set to 7.0–7.2 with Na_2_CO_3_. In order to precipitate large particles, the B-CD solution was centrifuged at 14000 rpm for 30 min. Finally, the solution was purified with dialysis tubing (MWCO 2000) for 6 h in ultrapure water (18.2 MΩ), and the water was renewed every 2 h. Some part of the purified solution was stored at 4 °C, and the other part was powdered by using a freeze-dryer for its characterization.

### 4.4 Fluorescence Measurements of B-CDs

The synthesized B-CDs were obtained to characterize their fluorescence behavior. In order to investigate the fluorescence behavior of B-CDs, the emission spectra at various excitation wavelengths, as well as the wavelength at the maximum value in the absorption spectra, were obtained. The quantum yields of B-CDs were calculated relative to tryptophan (Φ: 0.13) excited at 280 nm.^26^

### 4.5 Culturing of Colo 320 Cells

In this study, the primary human colon cancer cell line (COLO 320, HTL95027, INTERLAB Cell Line Collection, Genoa, Italy), which have semiadhesive cell morphology, was cultured in RPMI-1640 (F-1213, Biochrom, Berlin, Germany) containing 10% fetal bovine serum (Capricorn Scientific, FBS-12B), 1% l-glutamine (Capricorn Scientific, GLN-B), and 1% penicillin–streptomycine (Capricorn Scientific, PS-B) at 37 °C in an incubator (ESCO, CCL-170B-8) with 5% CO_2_ in air. Morphological imaging of the cells was performed with an inverted microscope with phase-contrast attachment (IX71, Olympus, Japan). The cells were cultured until 80% confluency was obtained.

### 4.6 Isolation of Colo 320 CD133+ Cells

CD133 was used as a colon cancer stem cell marker. The cells were collected in the culture medium after trypsinization with 0.5% trypsin–EDTA solution (Biochrom, L2133). The supernatant after centrifugation (5 min at 1000 rpm) was discarded, and the cells were collected in phosphate buffer solution containing 0.5% BSA and 0.08% EDTA (PBS-BE) and incubated with CD133IgGs (Miltenyi Biotech) on ice for 15 min. The cells were centrifuged again, the same as above, and resuspended in PBS-BE solution. The cell suspension was poured into the MiniMACs system column reservoir, and Colo 320 CD133– cells were collected in a 50 mL tube. Separated columns from MiniMacs stand were washed with PBS-BE solution to collect Colo 320 CD133+ cells. The cells were cultured in the culture medium, as given above until 80% confluence was obtained.

### 4.7 Culturing of Control Cells (Vero Cells)

Vero cells (African green monkey kidney, ATCC-CCL-81) were cultured in DMEM-F12 (Gibco, 31330-038) supplemented with 10% Fetal bovine serum (FBS, Capricorn Scientific, FBS-12B), 1% l-glutamine (Capricorn Scientific, GLN-B), and 1% penicillin–streptomycin (Capricorn Scientific, PS-B) in a humidified atmosphere at 37 °C and 5% CO_2_. The morphology of the cells was examined every second day using a phase-contrast inverted microscope (IX71, Olympus, Japan) and photographed. When the cells were confluent, they were routinely subcultured using 0.25% trypsin–EDTA (Biochrom, L2133) solution. For the immunohistochemistry assay, the cells were seeded on a 24-well plate (5 × 10^3^ cells in each well) including circled coverslips covered with FBS and cultured for 24 h for attachment. After that glutamine 190 or 230 or 270 °C was administrated which is explained in the main text Section 4.9.

### 4.8 Cytotoxicity Assay

Cytotoxicity effects of B-CDs synthesized at three different temperatures of 190, 230, and 270 °C were examined by MTT analysis. Colo 320 CD133+ and CD133– cells were seeded on a 96-well plate and every well included 5 × 10^3^ cells. They were cultured for 24 h at 37 °C and 5% CO_2_. The cells were incubated in different dilutions (1:1, 2:1, and 4:1) of all B-CDs for 24 and 48 h. For the control group, culture medium or untreated CD133+ and CD133– cells were also cultured at the same time as study groups. After 48 h, 0.5 mg/mL MTT solution (Glentham Life Sciences, 471OVO) was added to each well, and incubated for 4 h at 37 °C. At the end of the incubation time, 50 μL of dimethyl sulfoxide (DMSO, Sigma, D2650) was added to each well and measurements were performed at 450–690 nm absorbance spectrophotometry (BioTek Instruments Inc., ELX800UV). The analyses were conducted three consecutive times.

### 4.9 Immunohistochemistry

Distributions of caspase 3, Ki67, lamin B1, P16, and cytochrome *c* on Colo 320 CD133+ and CD133– cells were analyzed by the indirect immunoperoxidase technique. The cells were seeded on a 24-well plate (5 × 10^3^ cells in each well) including circled coverslips covered with FBS and cultured for 24 h for attachment. The cells were then incubated in 1:1 dilution of glutamine 190 or 230 or 270 °C for 24 h in a 37 °C incubator under a humidified atmosphere of 5% CO_2_. For control groups, Colo 320 CD133+ and CD133– cells were only cultured as the culture medium. After incubation time, all cells were fixed with 4% paraformaldehyde (pH 7.4 - Merck, TP704404-415) at room temperature for 30 min after washing with PBD Lonza, BE17-516F. The cells were also washed with PBS several times after fixation, and cells were then treated with 0.1% Triton-X-100 (AppliChem, A4977-0100) on ice for 15 min for permeabilization. After washing with PBS, 3% hydrogen peroxide (H_2_O_2_, Merck, K31355100 303) was added to cells for 10 min, after washing with PBS, blocking solution (Novex Life Technologies, 859,043) was added to the cells for 1 h at room temperature. After removing the blocking solution, primary antibodies anticaspase-3 (GeneTex, GTX78090), anti-*K*_i_-67 (NeoMarkers, RB-081-A1), antilamin B1 (Proteintech, 12987-I-AP), anti-P16 (Proteintech, 10883-I-AP), and anticytochrome *c* (Santa Cruz Biotechnology, sc-13156) were added and incubated overnight at +4 °C. Primary antibodies were discarded, and cells were washed with PBS and incubated with biotinylated secondary antibody and streptavidin-horseradish peroxidase (Novex Life Technologies, 859043), 30 min for every incubation. The cells were washed with PBS and diaminobenzidine (DAB, Millipore IHC Select, 71898) was kept for 5 min to visualize immunoreactivity. After being washed with distilled water, the cells were stained with Mayer’s hematoxylin for 1 min and washed with distilled water again. Coverslips were transferred onto slides and mounted with the mounting medium (Spring Bioscience, DMM-125), they were analyzed under a light microscope (BX43, Olympus), and positive and negative control staining were performed to specify immunoreactivity during immunocytochemistry. Experiments were performed three times for every group, and evaluations were performed by two independent researchers. Intensities of immunoreactivity were evaluated as negative (−), weak (+), moderate (++), or strong (+++).

## Supporting Information Available

Relationship between temperature and quantum yield (Figure S1), X-ray photoelectron spectra (Figure S2–S4), cell images (Figure S5–S7), and X-ray photoelectron spectra binding energy tables (Tables S1–S3) (PDF)
Raw data of FTIR, ζ potentials, XRD, TGA, XPS, UV–vis absorbance, photoluminescence, and cytotoxicity experiments (XLSX)

## Abbreviations

CD: carbon dot
B-CD: boron-doped carbon nanodot
FA: folic acid
FR: folate receptor
CNS: central nervous system
BBB: blood–brain barrier
XRD: X-ray diffraction
TEM: Transmission electron micrographs
XPS: X-ray photoelectron spectroscopy
PBA: phenylboronic acid
GLU: glutamine
PL: photoluminescence
