# Supplementary material for: Boron-Doped Carbon Nanodots as a Theranostic Agent for Colon Cancer Stem Cells
Source: ACS Omega. 2023 Aug 10;8(33):30285–93. doi: 10.1021/acsomega.3c03154 (PMC10448486; doi:10.1021/acsomega.3c03154)
Supplement: Supplementary file 1 — ao3c03154_si_001.pdf [file ao3c03154_si_001.pdf]

SUPPORTING INFORMATION

Boron doped carbon nanodots as a theranostic agent for colon cancer stem cells

Sezgin Ozkasapoglu[a], Mehmet Gokhan Caglayan[b], Fatih Akkurt[c], Hilal Kabadayi Ensarioğlu[d], H. Seda Vatansever[d,e], Huseyin Celikkan[f]**

[a] Turkish Nuclear Energy and Mineral Research Agency (TENMAK), Boron Research Institute (BOREN) Ankara, 06520, Turkey

[b] Ankara University, Faculty of Pharmacy, Department of Analytical Chemistry, Ankara, 06560,Turkey

[c] Gazi University, Faculty of Engineering, Department of Chemical Engineering, Ankara, 06570, Turkey.

[d] Manisa Celal Bayar University, Faculty of Medicine, Department of Histology and Embryology, Manisa, 45030, Turkey

[e] DESAM Institute, Near East University, Mersin 10, Turkey.

[f] Gazi University, Science Faculty, Department of Chemistry, Ankara, 06500, Turkey.

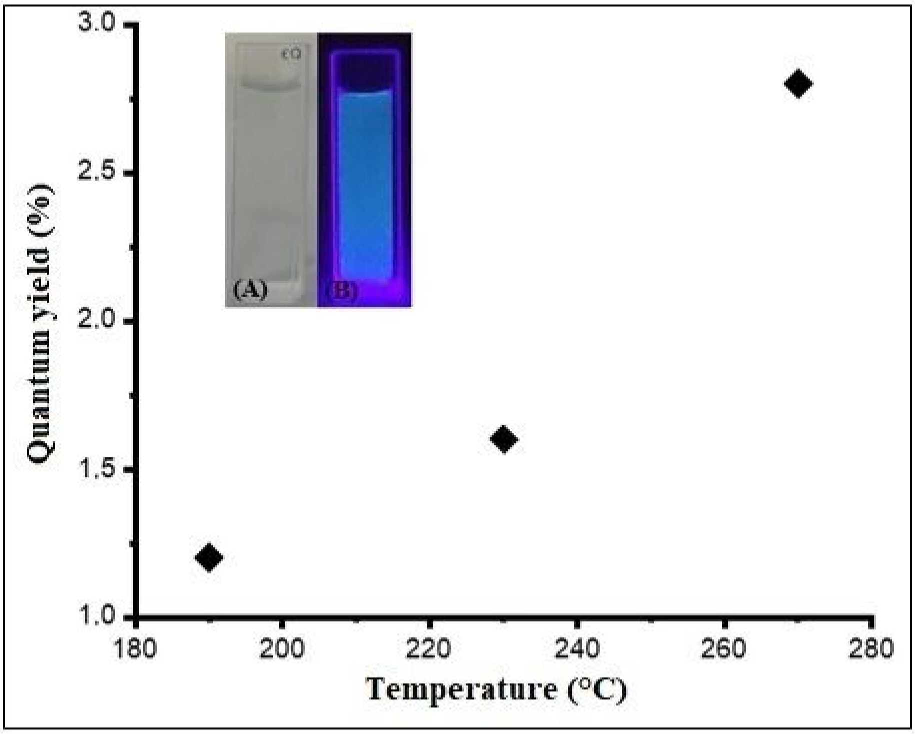

Figure S1. Relationship between temperature and quantum yield. (inset: the photograph of B-CDs at synthesized at 270 ^{o}C (A) under daylight (B) under 395 nm UV light)

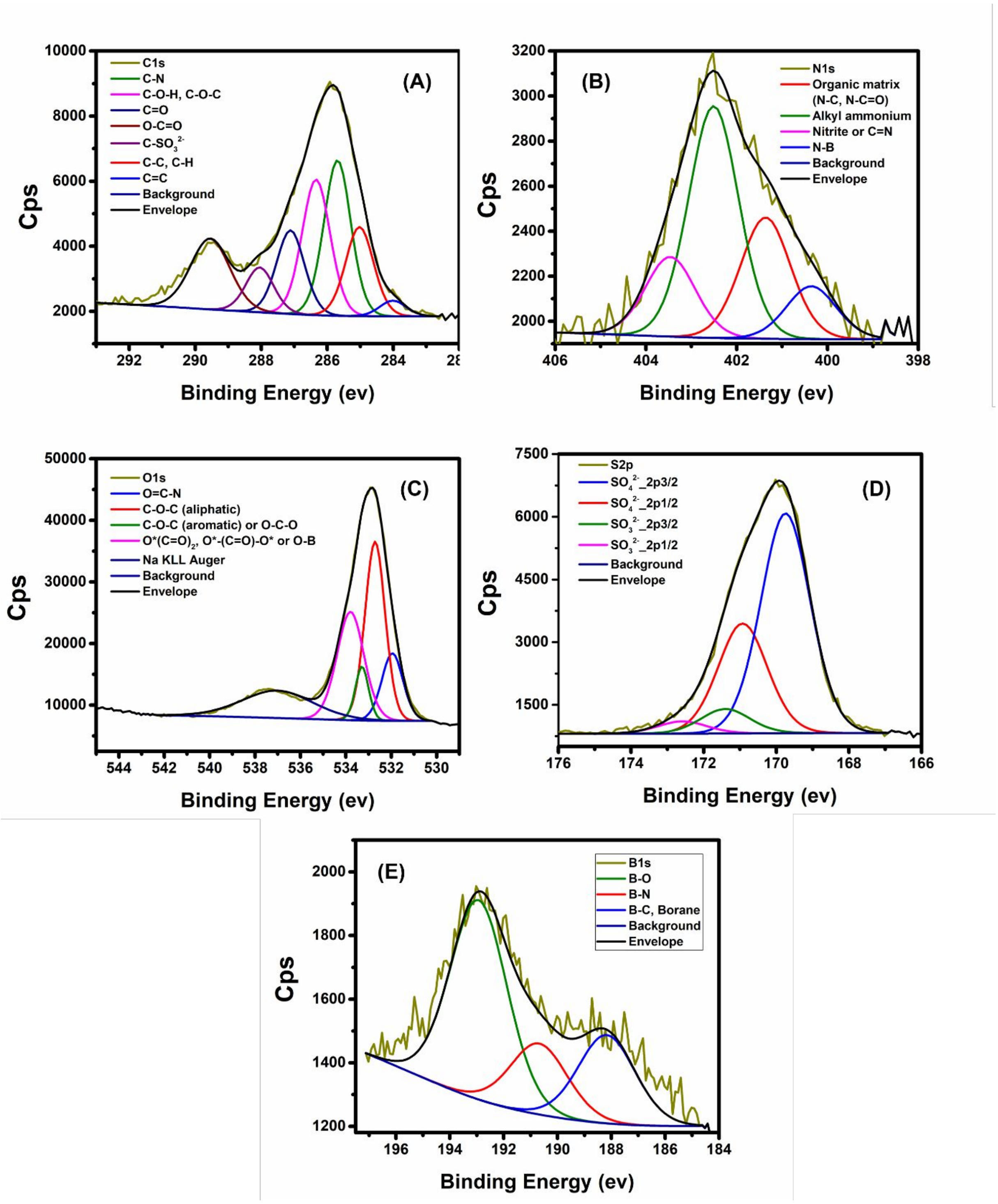

Figure S2. Binding energies of (A) C1s, (B) N1s, (C) O1s, (D) S2p and (E) B1s with their various chemical environments for B-CDs synthesized at 190 ^{o}C.

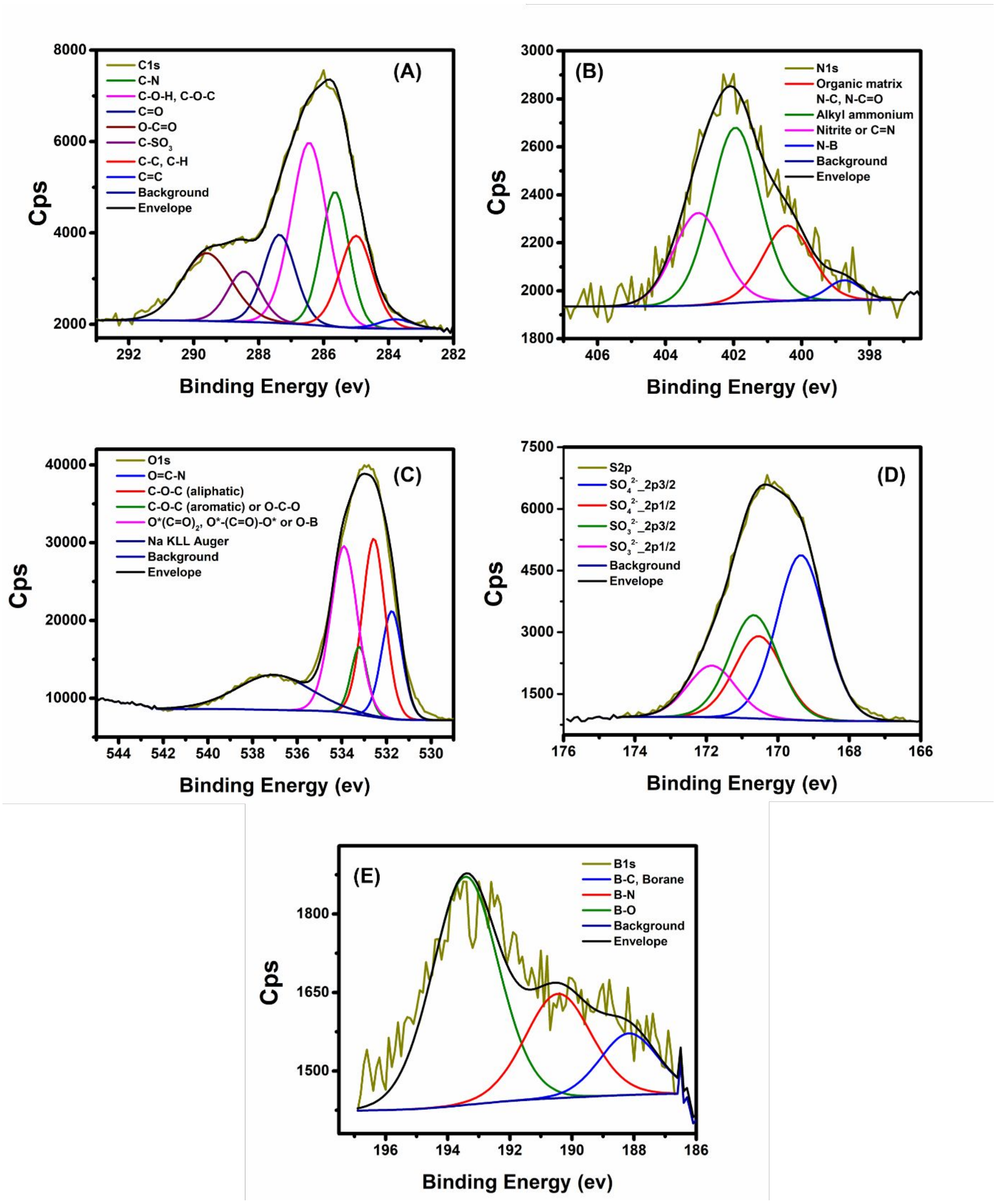

Figure S3. Binding energies of (A) C1s, (B) N1s, (C) O1s, (D) S2p and (E) B1s with their various chemical environments for B-CDs synthesized at 230 ^{o}C.

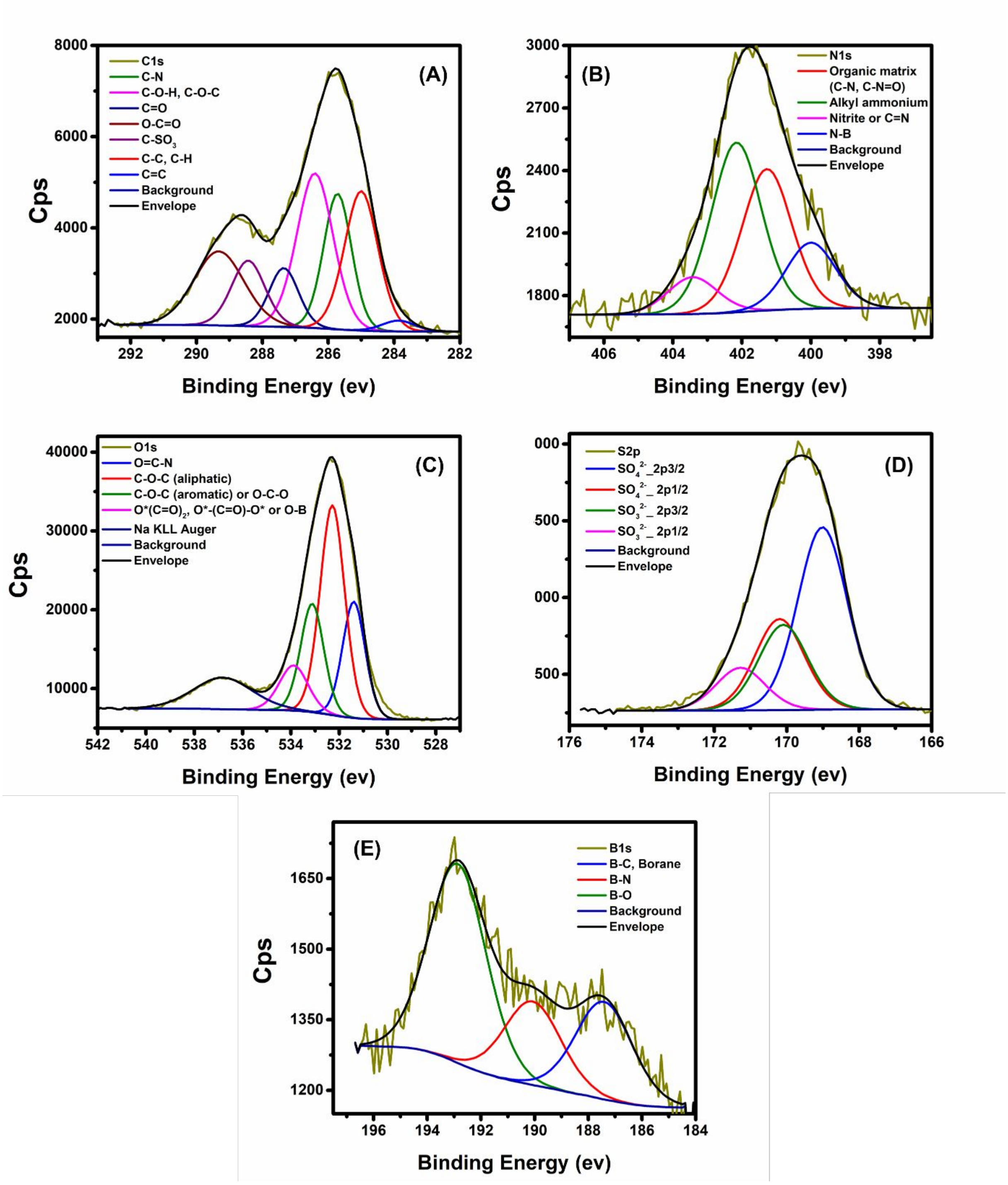

Figure S4. Binding energies of (A) C1s, (B) N1s, (C) O1s, (D) S2p and (E) B1s with their various chemical environments for B-CDs synthesized at 270 ^{o}C.

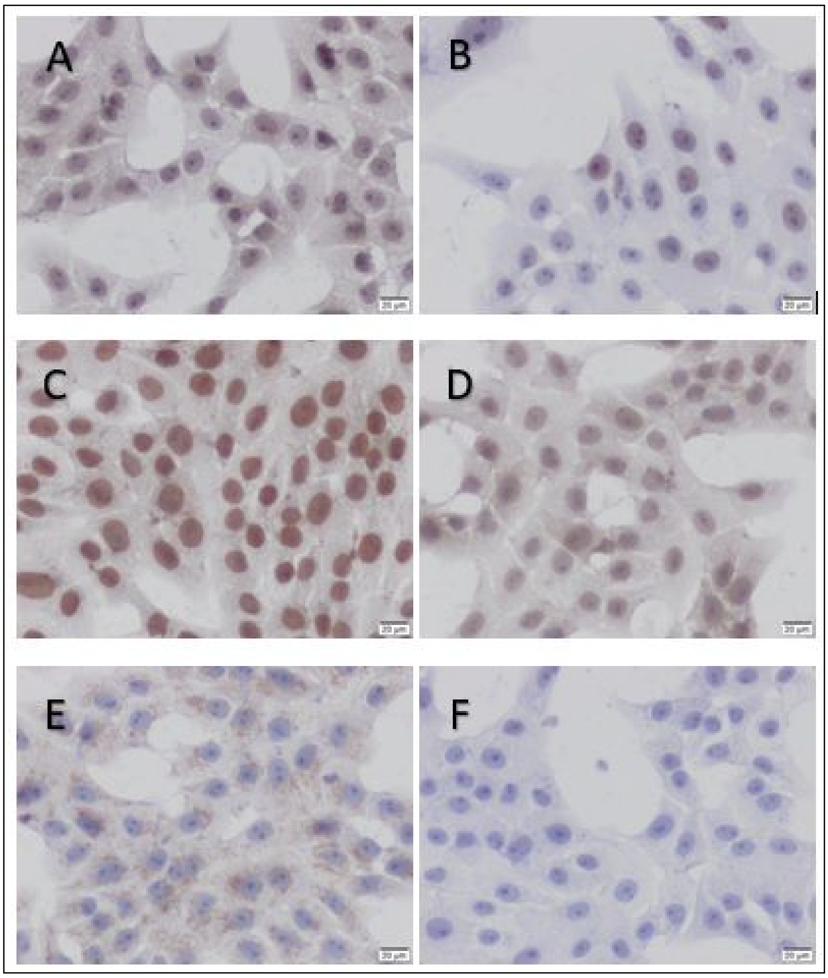

Figure S5. Caspase 3 (A), Ki67 (B), lamin B1 (C), P16 (D) and cytochrome C (E) immunoreactivity in Vero cells after 24 hours of B-CD administration synthesized at 190 °C. Control immunohistochemical staining (F). Scale bars: 20 μm.

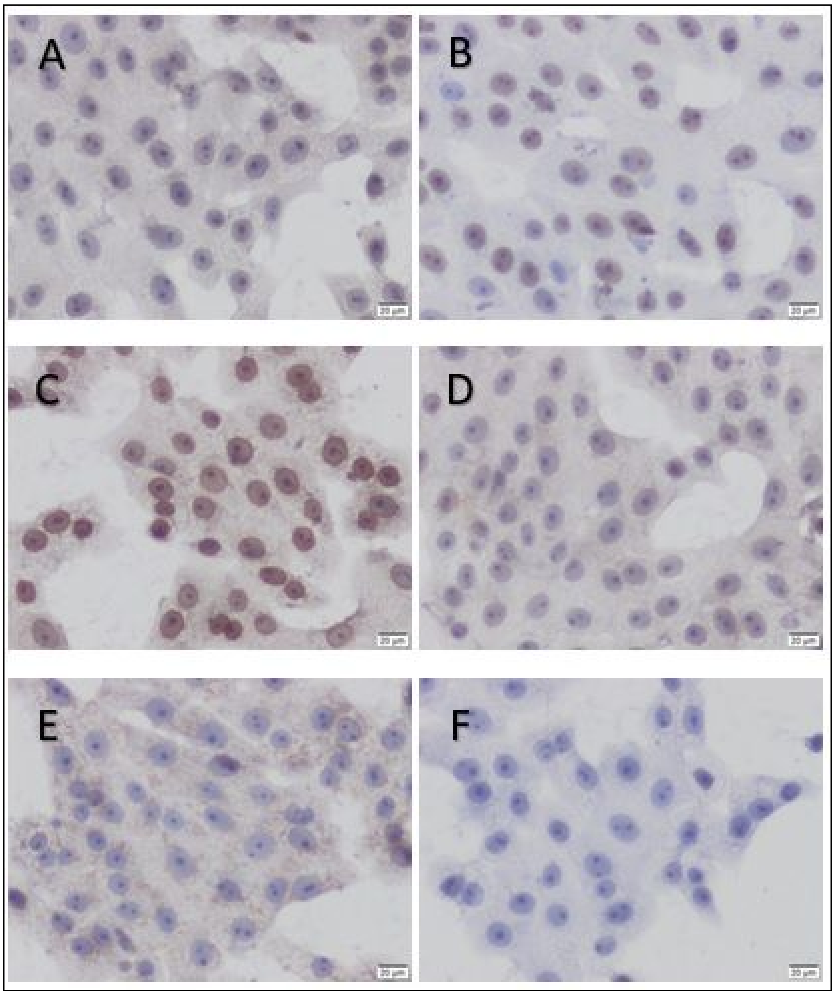

Figure S6. Caspase 3 (A), Ki67 (B), lamin B1 (C), P16 (D) and cytochrome C (E) immunoreactivity after 24 hours of B-CD administration synthesized at 230 °C in Vero cells. Control immunohistochemical staining (F). Scale bars: 20 μm.

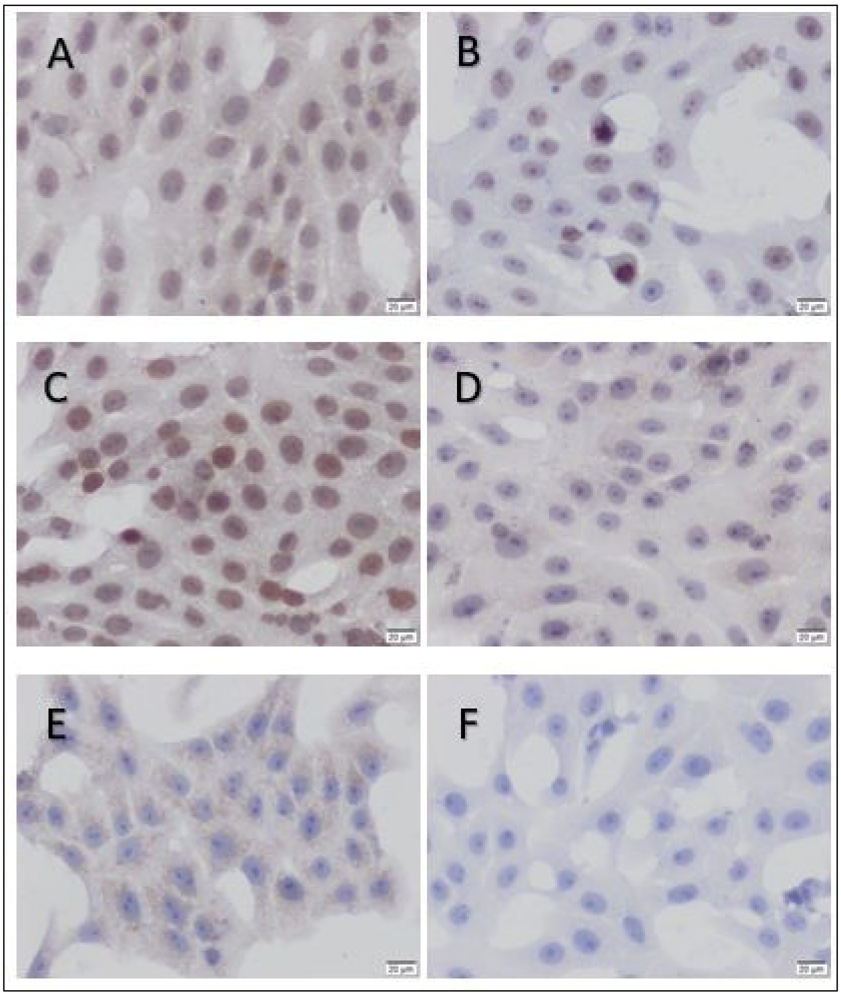

Figure S7. Caspase 3 (A), Ki67 (B), lamin B1 (C), P16 (D) and cytochrome C (E) immunoreactivity after 24 hours of B-CD administration synthesized at 270°C in Vero cells. Control immunohistochemical staining (F). Scale bars: 20 μm.

Table S1. Binding energies and percentages of N1s for B-CDs synthesized at 190 °C, 230 °C and 270 °C.

	**Synthesis temperature of B-CDs**					
**N1s**	**190 °C**		**230 °C**		**270 °C**	
	**B.E. (eV)**	**%**	**B.E. (eV)**	**%**	**B.E. (eV)**	**%**
N-B	400.4	10.9	398.8	3.81	400.1	16.0
Organic matrix (N-C, N-C=O)	401.4	25.0	400.5	20.9	401.4	34.1
Alkyl ammonium	402.5	47.7	402.0	49.3	402.3	41.0
Nitrite or C=N	403.5	16.4	403.1	26.0	403.5	8.91

Table S2. Binding energies and percentages of O1s for B-CDs synthesized at 190 °C, 230 °C and 270 °C.

O1s	Synthesis temperature of B-CDs 190 °C		230 °C		270 °C	
	B.E. (eV)	%	B.E. (eV)	%	B.E. (eV)	%
O=C-N	532.0	13.5	531.8	15.6	531.5	19.5
C-O-C (Aliphatic)	532.7	33.9	532.6	28.1	532.4	38.4
C-O-C (aromatic) or O-C-O	533.3	6.73	533.3	7.80	533.2	18.7
O*-(C=O)$_2$, O*-(C=O)-O* or O-B	533.8	27.5	534.0	30.6	534.0	9.64
Na KLL Auger	537.0	18.4	537.1	17.9	536.9	13.9

Table S3. Binding energies and percentages of S2p for B-CDs synthesized at 190 °C, 230 °C and 270 °C.

S2p	Synthesis temperature of B-CDs 190 °C		230 °C		270 °C	
	B.E. (eV)	%	B.E. (eV)	%	B.E. (eV)	%
SO_4^{2-}, S2p1/2	170.9	30.0	170.5	20.5	170.2	22.7
SO_4^{2-}, S2p3/2	169.8	60.0	169.4	41.0	169.0	45.42
SO_3^{2-}, S2p1/2	172.6	3.34	171.9	12.8	171.3	10.6
SO_3^{2-}, S2p3/2	171.4	6.70	170.7	25.7	170.1	21.2
